# Nanophotonics in Molecular Imaging and Biomedicine: Diagnostics, Therapies, and Translational Challenges

**DOI:** 10.1007/s11307-025-02061-w

**Published:** 2025-11-17

**Authors:** Youssef M. Hassan, Ahmed Wanas, Ayat A. Ali, Wael M. El-Sayed

**Affiliations:** 1 Department of Zoology, Faculty of Science, Ain Shams University, Abbassia, Cairo, 11566 Egypt; 2 Department of Biochemistry, Faculty of Science, Ain Shams University, Abbassia, Cairo, 11566 Egypt; 3 Biotechnology Program, Faculty of Science, Ain Shams University, Abbassia, Cairo, 11566 Egypt

**Keywords:** Lab-on-a-chip, Machine learning, Optical biosensing, Personalized medicine, Photothermal therapy, Surface plasmon resonance

## Abstract

Nanophotonics—the manipulation of light at the nanometer scale within biological systems—is transforming molecular imaging and photobiology, enabling advanced *in vivo* imaging, diagnostics, and therapy guidance. This review outlines core nanophotonic principles, including surface plasmon resonance, optical confinement, and photon–matter interactions, underpinning emerging molecular imaging probes and diagnostic tools. Biocompatible nanomaterials such as quantum dots, gold nanoparticles, and photonic metamaterials enable highly sensitive, selective imaging and biosensing for early, minimally invasive disease detection and monitoring. Targeted photothermal and photodynamic therapies using near-infrared (NIR) and NIR-II light advance image-guided interventions, allowing deeper tissue penetration with minimal collateral damage. We also discuss the integration of nanophotonic components into lab-on-a-chip and microfluidic platforms for point-of-care diagnostics, accelerating clinical translation. Additionally, machine learning enhances molecular imaging analysis and probe optimization, enabling real-time data interpretation and predictive modeling tailored to patient-specific profiles. This article is a narrative review that emphasizes recent advancements from 2021–2025, identified through targeted database searches, highlighting progress, research gaps, and future perspectives for disease-specific applications. While these advances hold promise, challenges remain in biocompatibility, light penetration, scalability, and regulatory approval. Collectively, integrating nanophotonics with molecular imaging, machine learning, and personalized medicine frameworks marks a step toward next-generation precision diagnostics and image-guided therapeutics.

**Supplementary Information:**

The online version contains supplementary material available at 10.1007/s11307-025-02061-w.

## Methodology

This narrative review synthesizes recent advancements in nanophotonics for biomedical applications, focusing on developments from 2021 to 2025. A targeted literature search was conducted in PubMed, Scopus, and Web of Science for English-language, peer-reviewed studies on nanophotonic biosensing, bioimaging, photothermal therapy, drug delivery, and diagnostics within experimental and translational contexts. Boolean operators (AND, OR) were applied to refine retrieval using keywords such as “nanophotonics,” “plasmonics,” “metasurfaces,” “surface-enhanced Raman scattering,” “SERS,” “photothermal therapy,” “bioimaging,” and “biosensors.” Approximately 510 articles were initially retrieved, of which 63 were selected and cited in this manuscript. Original research articles were prioritized, while review papers were included selectively to provide background or when they contained comprehensive data not available in primary sources. Exclusion criteria included non-English publications, non-peer-reviewed materials, and studies outside biomedical applications.

Priority was given to studies with experimental validation, translational insights, and emerging healthcare technologies, while theoretical works lacking biomedical relevance were generally excluded. To minimize bias, studies from diverse groups and regions were included, though restricting to English-language publications may have omitted some regional advances. This approach provides a cohesive synthesis of principles, progress, and future directions in nanophotonics with translational and clinical relevance.

## Introduction

Nanophotonics, the study of light at the nanometer scale, enables control of optical fields beyond diffraction limits, allowing near-field coupling, photonic bandgap (PBG) effects, and localized surface plasmon resonance (LSPR). Such capabilities permit nanostructures to confine and manipulate light with high precision, offering powerful biomedical tools for diagnosis, monitoring, and therapy [1].

Initially rooted in quantum optics, materials science, and nanofabrication, the field advanced through metal nanoparticles that enhance electromagnetic fields [2]. Integration with life sciences expanded applications to light-activated therapies, nanoparticle-based imaging, and biosensing, supporting precision-guided photothermal and photodynamic treatments, early cancer detection, and pathogen identification [3].

Nanostructured photonic crystals and metamaterials now enable label-free, real-time biosensing with ultrahigh sensitivity, while gold nanorods and quantum dots (QDs) facilitate deep-tissue *in vivo* imaging [4]. Recently, clinical physicians and scientists in the field of medical materials have shown strong interest in nanoprobes because of their relatively precise diagnostic and therapeutic capabilities as well as their ability to enable noninvasive *in vivo* imaging. Demonstrated applications include the detection of atherosclerotic plaque, gastric acid, silicon and silica materials, and even adenosine triphosphate at the single-cell and subcellular levels, offering new possibilities for precision medicine. With nanoprobes, significant advancements have been made in live imaging, and research in this area is ongoing [5]. It is important to note, however, that most of these nanoprobe-based *in vivo* imaging approaches remain at the preclinical stage, whereas other nanophotonic technologies such as lateral flow assays and SPR-based biosensors are already established in clinical diagnostics. Despite these advances, challenges remain—particularly probe accumulation in the body and uncertainties regarding clearance via the kidney or liver. Characterizing physicochemical features, pharmacodynamics, pharmacokinetics, process control, biocompatibility, and nanotoxicity, as well as addressing scale-up and reproducibility, remain essential for translation. These difficulties reflect the differing demands of patients (clinical and therapeutic use), industry (production), and regulatory bodies (authorization processes), and highlight the need for coordinated solutions to accelerate clinical adoption [6].

Additionally, optogenetics and light-mediated cellular control have expanded nanophotonics into regenerative medicine [7]. Yet challenges persist, including cytotoxicity, limited light penetration, production scalability, and regulatory uncertainties [8]. Many applications remain preclinical, requiring solutions for effective translation into medical practice.

LSPR and surface plasmon–polaritons underpin many biomedical applications by enhancing light–matter interactions at subwavelength scales. Metallic nanoparticles generate intense electromagnetic fields for sensing, imaging, SERS, and energy conversion, with noble metals supporting refractive index sensing and luminescence modulation [9]. Gold nanoparticles (AuNPs) exhibit strong LSPR, amplifying photoluminescence, Raman scattering, and nonlinear effects. Geometries such as nanotetrahedrons and closely spaced dimers create “hot spots” with high enhancement factors. In SERS, AuNPs boost weak Raman signals via chemical and electromagnetic mechanisms, serving as highly effective substrates [10].

Machine learning (ML), particularly deep learning (DL), is transforming nanophotonic device design. While early devices like split-ring resonators were manually optimized, rising complexity now demands computational approaches. DL models trained on simulation datasets predict and optimize device performance, accelerating development of next-generation biomedical systems. Remaining challenges include the need for extensive training data and risks of approximation errors [11, 12].

This review critically examines nanophotonics in biomedical applications with a focus on modality-specific translational barriers—including optical property variability driven by nanoscale size, shape, and surface chemistry; protein-corona formation and RES sequestration that alter pharmacokinetics and long-term retention; practical constraints of *in vivo* light delivery and dosimetry (e.g., NIR/NIR-II penetration, thermal/phototoxic limits); and the regulatory complexity of multifunctional platforms that couple materials with illumination hardware. We introduce foundational principles of nanoscale light–matter interactions, analyze recent advances in imaging, diagnostics, therapy, and tissue engineering, and outline engineering approaches and nanophotonics-specific regulatory strategies tailored to these challenges, while mapping emerging trends relevant to clinical translation.

## Fundamentals of Nanophotonics

### Light–Matter Interaction at the Nanoscale

At nanometer dimensions, optical behavior deviates from classical optics, requiring a framework that integrates Maxwell’s electromagnetic theory with quantum mechanics. Confinement of photons and electrons in nanoscale structures generates unique phenomena such as near-field effects, quantum confinement, and discrete energy levels, which are inaccessible in bulk systems [13].

Near-field interactions, mediated by evanescent waves that decay exponentially from a surface, enable highly localized energy transfer. These localized fields amplify the cross-section of light–matter interactions, thereby enhancing detection sensitivity and enabling imaging beyond the diffraction limit [14]. Such effects form the physical basis for nanophotonic biosensing and bioimaging.

Metallic nanostructures exhibit surface plasmon resonance (SPR)—collective oscillations of conduction electrons at the metal–dielectric interface. This resonance produces intense, localized electromagnetic fields that greatly amplify optical signals. A related phenomenon, localized surface plasmon resonance (LSPR), occurs in nanoparticles and provides tunable optical properties depending on size, shape, and composition. These plasmonic effects are central to label-free biosensing, real-time molecular detection, and nanoscale imaging [15].

In semiconductor nanocrystals and quantum dots (QDs), quantum confinement restricts electron–hole pairs, creating discrete, size-dependent energy levels. This property gives rise to bright, photostable, and spectrally tunable fluorescence, which is highly advantageous for multiplexed imaging of biomolecules. Furthermore, energy transfer mechanisms such as Förster resonance energy transfer (FRET) enable nanometer-scale readouts of biomolecular interactions. By monitoring changes in fluorescence based on spectral overlap and dipole orientation, FRET provides real-time information on DNA hybridization, protein folding, and receptor–ligand binding dynamics [16].

Together, these nanoscale optical phenomena establish the foundation of nanophotonics in biology by offering enhanced sensitivity, spatial resolution, and multiplexing capacity for molecular detection and imaging.

### Surface Plasmon Resonance (SPR)

**Fig. 1:**
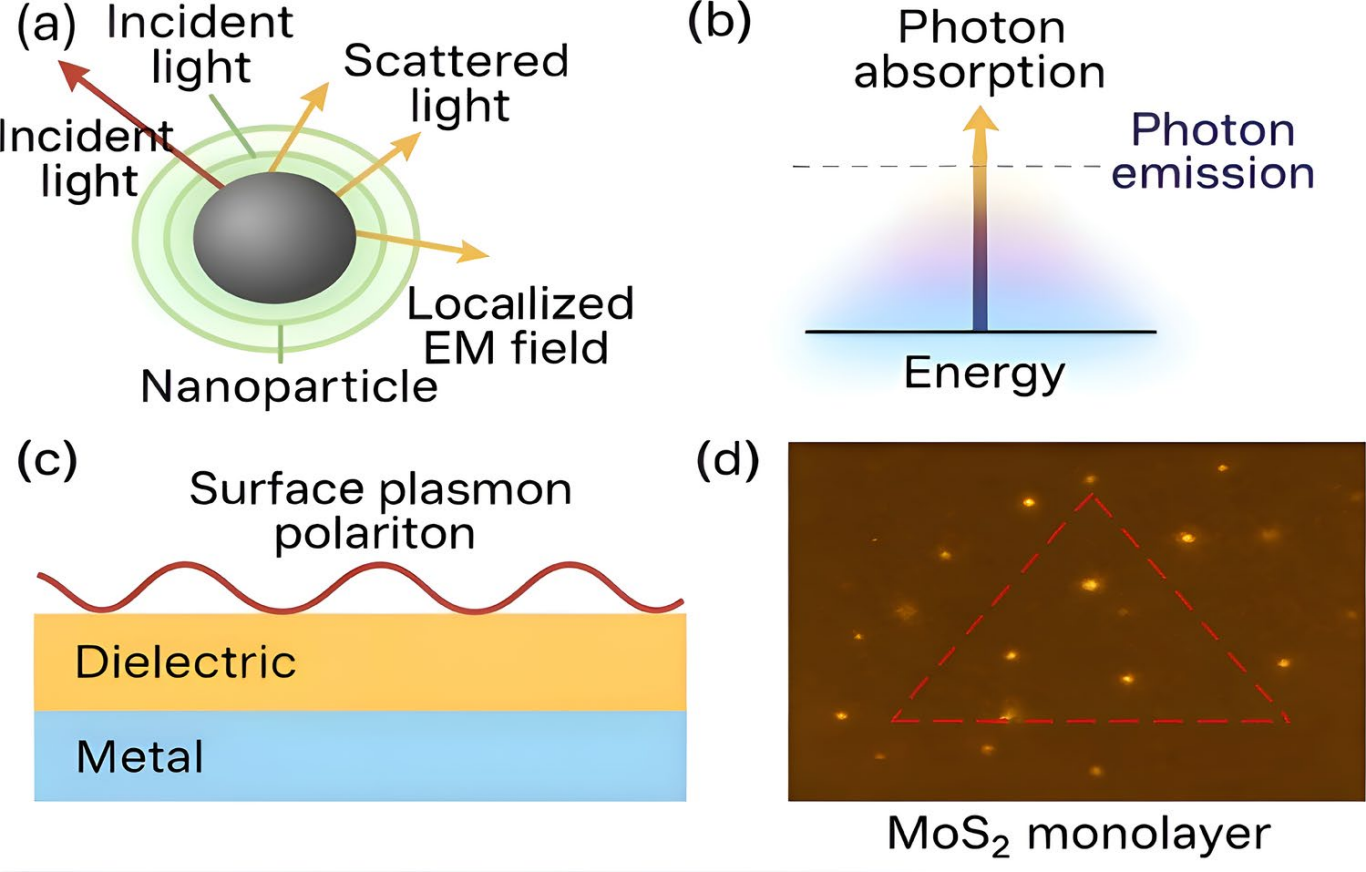
(**a**) Light–nanoparticle interaction generating localized electromagnetic (EM) fields, which form the basis of surface plasmon resonance (SPR) sensing. (**b**) Schematic illustration of photon absorption and emission processes in nanophotonic systems. (**c**) Surface plasmon polariton (SPP) propagation along a metal–dielectric interface. (**d**) Dark-field image of silver (Ag) nanocubes on molybdenum disulfide (MoS₂), demonstrating strong plasmon–exciton interactions

SPR is one of the most widely applied nanophotonic principles in biomedicine. It arises when incident light couples with free electrons at a metal–dielectric interface, producing resonance that is exquisitely sensitive to changes in the local refractive index [17]. This sensitivity allows real-time, label-free detection of molecular interactions, including protein–protein binding, nucleic acid hybridization, and drug–target interactions (Fig. 1).

Modern SPR platforms integrate advanced computational modeling to optimize sensor performance. Approaches such as Finite-Difference Time-Domain (FDTD) and Finite Element Method (FEM) simulations predict how sensor geometry and material selection influence resonance sensitivity [18]. These computational tools accelerate device design, enabling high-performance plasmonic biosensors for biomedical diagnostics and pharmacological screening.

### Plasmonic Nanoparticles

Noble metal nanoparticles, particularly gold (AuNPs) and silver (AgNPs), play a central role in nanophotonics due to their ability to sustain LSPR. When illuminated, these nanoparticles generate localized electromagnetic fields that amplify optical signals in their immediate vicinity. This makes them powerful tools for surface-enhanced Raman spectroscopy (SERS), plasmon-enhanced fluorescence, and photothermal imaging [19].

The versatility of AuNPs stems from their size- and shape-dependent tunability, high surface-to-volume ratio, and chemical stability. For example, anisotropic structures such as nanorods and nanostars exhibit resonances in the near-infrared (NIR) region, which penetrates biological tissues, enabling deep-tissue imaging and targeted photothermal therapy [20].

In biosensing, AuNPs are widely used in both colorimetric and spectroscopic detection schemes. In colorimetric assays, analyte-induced aggregation of AuNPs alters their plasmon coupling, leading to visible color changes. These simple assays are highly attractive for point-of-care applications. Alternatively, immobilized nanoparticles on solid supports can generate stable, reproducible LSPR signals that are quantified using UV–Vis spectroscopy [21]. Such platforms allow sensitive, real-time detection of biomolecules, ions, and pathogens in clinical samples.

PCR is the gold standard for nucleic acid detection but is limited in POC settings by the size, cost, and slow heating rates (~ 1–3 °C/s) of conventional systems, as highlighted during the COVID-19 pandemic. While qPCR enables real-time monitoring, it adds complexity to miniaturization [22].

To address these limitations, gold nanorods (AuNRs) have been employed to convert NIR (850 nm) light into localized heat, enabling rapid thermal cycling (~ 10–15 °C/s) with compact optics. In plasmonic PCR, AuNRs within thin-walled PCR tubes are illuminated by IR LEDs, producing fast, localized heating without interfering with fluorescence detection due to non-overlapping spectra. Cooling is fan-assisted and monitored via a thermocouple with LabVIEW control [22].

**Fig. 2:**
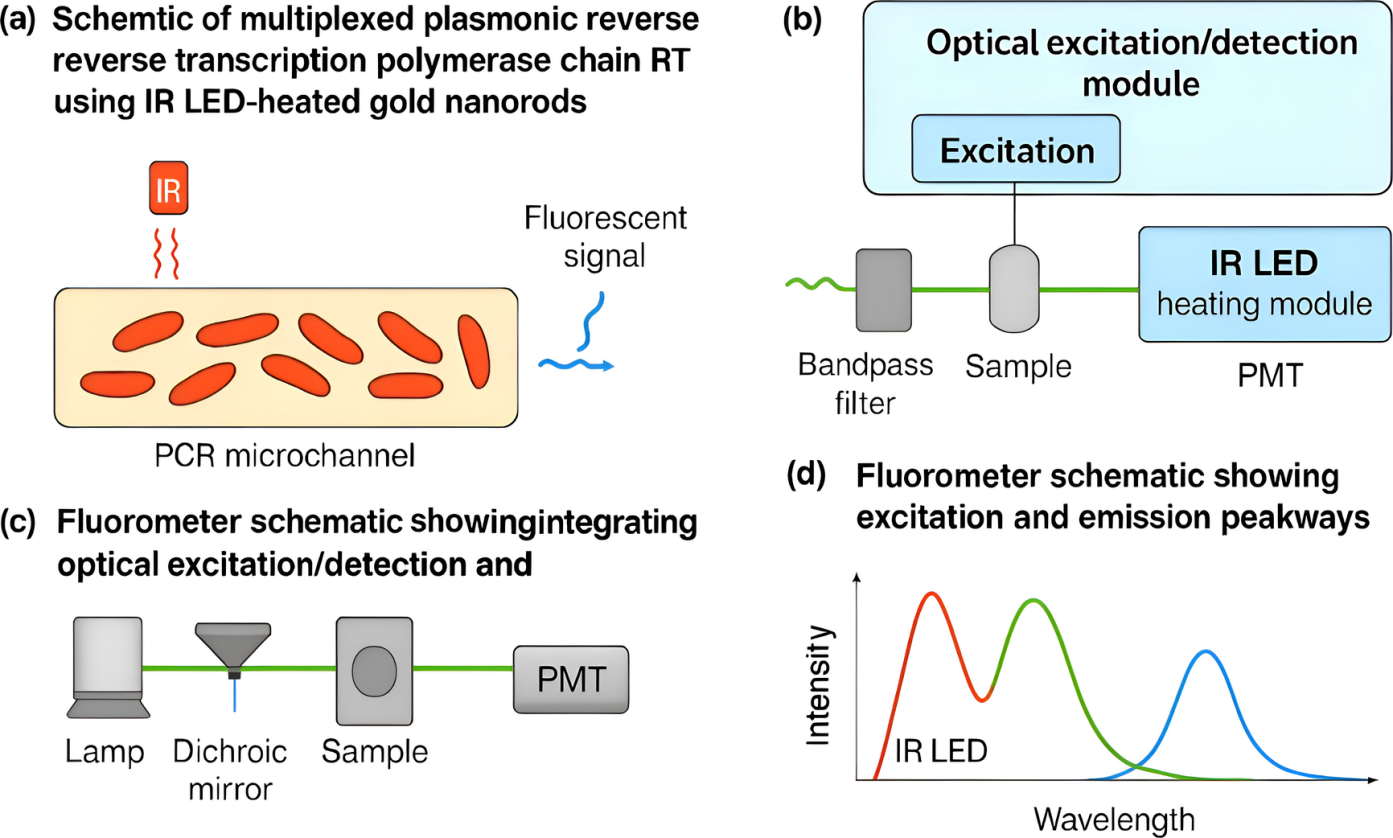
(**a**) Schematic of multiplexed plasmonic reverse transcription polymerase chain reaction (RT-PCR) using infrared light-emitting diode (IR LED)-heated gold nanorods (AuNRs) for rapid thermal cycling and real-time fluorescence detection. (**b**) Layout of a low-cost instrument integrating optical excitation/detection and IR LED heating modules. (**c**) Fluorometer schematic showing excitation and emission pathways. (**d**) Spectral profile illustrating component spectra and fluorescence emission peaks

This setup enables full RT-qPCR with multiplexed fluorescence per cycle, overcoming endpoint or single-wavelength constraints **(**Fig. 2**)**. It achieved accurate SARS-CoV-2 detection without cross-reactivity with related coronaviruses at high viral loads while preserving internal control signals [23].

Using clinical saliva samples without RNA extraction, the platform delivered 100% sensitivity and specificity compared to lab-based PCR within 22–23 min. Integration of AuNR-driven rapid heating and compact fluorescence optics supports real-time, multiplexed SARS-CoV-2 RNA detection with low detection limits (2.2–4.4 copies/μL) and reproducible performance across AuNR concentrations [24, 25]. This demonstrates a portable, precise molecular diagnostic system, highlighting nanophotonics’ potential for decentralized healthcare.

### Photonic Crystals

Photonic crystals are periodic dielectric structures with PBGs that block light propagation at specific frequencies. Bandgap engineering enables confinement and guidance of light, supporting strong light–matter interactions for biosensing. Two-dimensional photonic crystal slabs integrated with microfluidics have achieved real-time, label-free biomolecule detection with sub-nanomolar sensitivity. Photonic crystals can be fabricated in 1D, 2D, or 3D by alternating high- and low-dielectric zones [26].

A notable example is a simple colorimetric protease sensor based on porous silicon 1D photonic crystals, eliminating the need for spectrometric readout [27]. Here, a porous silicon rugate filter modified with methyl groups becomes hydrophobic, preventing water penetration. Protease cleavage of an immobilized hydrophobic protein releases fragments into the pores, raising the refractive index and producing a visible color shift. Detection reached 7.2 pmol without instrumentation, with no added benefit from spectral analysis.

Further adaptations used stimuli-responsive hydrogels within the porous matrix to detect hydrophilic proteins [28, 29]. Analyte-driven changes in hydrogel cross-linking density generated visible signals, enabling real-time, equipment-free biosensing. Porous silicon-based photonic crystals have also detected volatile organic compounds at parts-per-billion levels via refractive index modulation [30]. More recently, dynamic photonic crystals incorporating stimuli-responsive hydrogels have yielded adaptive optical sensors that shift structural color in response to environmental changes [31].

These developments establish photonic crystals as sensitive, real-time, low-cost tools for biomolecular and environmental monitoring.

### Quantum Dots and Energy Transfer Mechanisms

Quantum dots (QDs) are fluorescent semiconductor nanocrystals that combine high brightness, resistance to photobleaching, and size-dependent emission spectra. These properties make QDs superior to conventional dyes for multiplexed and long-term bioimaging. By adjusting their diameter, QDs can be tuned to emit across the visible and near-infrared spectrum, facilitating simultaneous imaging of multiple biomolecular targets in living systems [32].

Beyond direct fluorescence imaging, QDs are powerful donors and acceptors in FRET-based assays. FRET efficiency is extremely sensitive to molecular distances in the range of 1–10 nm, making it a “nanoscale ruler” for probing biological processes. QD–FRET systems have been used to monitor nucleic acid hybridization, detect single-base mismatches, and visualize conformational changes in proteins with high temporal resolution [16].

These mechanisms highlight how quantum confinement and energy transfer can be exploited for dynamic molecular sensing and high-resolution imaging in biology.

### Integration in Molecular Imaging and Biosensing

The convergence of plasmonics and semiconductor nanophotonics has transformed molecular biology and medical diagnostics. Plasmonic nanoparticles provide ultrasensitive, label-free detection of biomolecular interactions, while QDs enable multiplexed, long-term, and high-contrast imaging. Together, they extend the resolution and sensitivity of existing diagnostic methods, supporting early disease detection, single-cell analysis, and real-time monitoring of biomolecular events [33].

By focusing on these core nanophotonic mechanisms—light–matter interactions, plasmonic resonances, and quantum confinement—this section establishes the technological foundation for their application in molecular imaging and biology [34].

### Quantum Dots

QDs are semiconductor nanocrystals exhibiting quantum confinement, providing size-tunable emission wavelengths, high quantum yields, and superior photostability. These properties make QDs suitable for multiplexed fluorescence imaging, light-emitting devices, and biosensing. Despite concerns regarding the cytotoxicity of heavy-metal-based QDs (e.g., CdSe, PbS), the development of non-toxic carbon-based and silicon QDs has expanded their biomedical potential.

NIR-emitting graphene QDs have enabled high-resolution intravital imaging with long circulation times and renal clearance [35]. Dual-functional QDs combining fluorescence imaging with reactive oxygen species (ROS) generation have facilitated integrated diagnostics and photodynamic therapy [36]. Carbon quantum dots (CQDs) exhibit strong UV absorption (230–400 nm) with visible-range tails, arising from π-π transitions (260–320 nm) and n-π transitions (270–390 nm). These optical properties can be tuned via surface functionalization and passivation, enhancing quantum yields and shifting emission peaks.

For example, EDC passivation increased QY from 18.83% to 41.10%, while heteroatom doping (e.g., nitrogen, sulfur, phosphorus) further tuned emission wavelengths into the NIR and improved QY up to 78% for ZnS-doped CQDs. Synthesis conditions, including solvent choice and alkali content, also influence particle size and fluorescence properties, with higher NaOH concentrations yielding larger particles and red-shifted emissions [37].

While these tunable properties and enhanced biocompatibility position QDs as versatile platforms for high-sensitivity biosensing, targeted imaging, and theranostics, several limitations must be acknowledged. Many reports rely on small-scale laboratory syntheses, and reproducibility across different preparation methods remains inconsistent, with batch-to-batch variability affecting optical properties and biocompatibility. Long-term toxicity remains insufficiently characterized, particularly for heavy-metal-based QDs, as most studies focus on short-term *in vitro* models rather than comprehensive *in vivo* assessments. Moreover, although carbon- and silicon-based QDs offer improved safety profiles, their scalability and regulatory acceptance for clinical use remain uncertain. These challenges underscore the need for standardized synthesis protocols, rigorous toxicological evaluation, and translational studies before QDs can achieve widespread biomedical adoption.

### Metamaterials

Metamaterials are engineered composites with electromagnetic responses absent in natural media, enabling negative refraction, superlensing, and cloaking. By controlling light at subwavelength scales, they support sub-diffraction imaging, wavefront shaping, and enhanced absorption for nanophotonic applications [28, 36].

Integration of 2D materials such as graphene and TMDs has enabled tunable optical systems. Structures like metal/dielectric multilayers or metal nanowire arrays induce extreme anisotropy, with dielectric tensor components of opposite signs producing hyperbolic dispersion. These hyperbolic metamaterials exhibit isofrequency contours that allow all-angle negative refraction of the Poynting vector despite positive refractive index, enabling sub-diffraction imaging, compact photonic devices, and enhanced sensing [38, 39]. Their engineered responses position metamaterials as strong candidates for biosensing platforms demanding high-resolution imaging and detection sensitivity.

### Optical Nanoantennas

Optical nanoantennas convert propagating waves into localized energy, enhancing excitation and emission rates for single-molecule spectroscopy, fluorescence, and nanoscale photodetection [36, 37]. Designs such as Yagi–Uda and bowtie antennas achieve directional emission with sub-10 nm confinement, while DNA origami-based assembly provides nanometer precision for optimized field enhancement and programmable structures. Asymmetric antennas enable chiral optical responses, supporting enantiomer-selective detection [40]. Integration with Complementary Metal–Oxide–Semiconductor (CMOS)-compatible circuits is advancing on-chip sensing and communication.

DNA nanotechnology has expanded nanoantenna fabrication from simple motifs to dynamic, stimuli-responsive gigadalton-scale superstructures. DNA origami enables programmable 1D, 2D, and 3D templates for functional nanophotonic assemblies applied in biosensing, drug delivery, and synthetic biology. Recent reviews highlight the role of DNA-origami assemblies in nanophotonics and nanomedicine, positioning nanoantennas as key to next-generation miniaturized biosensing and photonic devices [40, 41].

## Nanophotonic Materials and Platforms: Metallic Nanostructures

Metallic nanostructures are central to nanophotonics due to their ability to manipulate light below the diffraction limit via localized surface plasmon resonance (LSPR), where conduction electrons collectively oscillate in resonance with incident light**.** LSPR generates intense electromagnetic fields confined to the nanostructure’s surface, enhancing light–matter interactions and enabling applications in bioimaging, biosensing, and image-guided therapies [20, 21, 42]. These plasmonic effects support techniques such as surface-enhanced Raman spectroscopy (SERS), plasmon-enhanced fluorescence, and photothermal imaging, all of which provide high sensitivity and spatial resolution for molecular detection in biological environments.

Recent advances in synthesis strategies have improved the performance and applicability of metallic nanostructures for imaging. Conventional chemical and physical methods can yield nanoparticles with tunable optical properties, but they often involve harsh reagents and conditions that limit biocompatibility. In contrast, bioinspired and biomimetic synthesis approaches—drawing from natural biomineralization processes—enable the formation of metallic nanostructures (e.g., Au, Ag, Pt, Pd, and metal oxides) under mild, environmentally friendly conditions [42]. These strategies employ biological systems (microorganisms, plants, peptides, and DNA) to guide nucleation and growth, producing nanoparticles with intrinsic functional groups that facilitate bioconjugation and improve stability in biological fluids [42].

Biotemplated synthesis, for example, uses biomolecules such as DNA, proteins, and polysaccharides as scaffolds to direct nanoparticle morphology. Functional groups including carboxyls, amines, and thiols stabilize metal ions and promote controlled nucleation, avoiding the need for toxic surfactants [42]. DNA’s predictable secondary structure and strong affinity for metal ions allow for precise control over nanoparticle geometry—yielding rods, cubes, spheres, or even nanowires. Such control is directly relevant for imaging applications, since LSPR wavelength and field confinement depend sensitively on particle size and shape [20]. Furthermore, atomically precise photoluminescent metal nanoclusters (< 2 nm) produced through biomolecular templating provide bright, stable emission useful for fluorescence and multimodal bioimaging [21, 43].

These bioinspired and biotemplated methods are therefore not presented as synthetic novelties alone, but as enabling tools that yield biocompatible and morphologically optimized metallic nanostructures tailored for imaging performance. By integrating tunable plasmonic properties with inherent biological compatibility, these approaches support the development of next-generation imaging probes that combine strong optical responses with safe *in vivo* applicability [22, 43]. Thus, bioinspired synthesis is discussed here not as a synthetic novelty, but as an enabling route to generate biocompatible and morphologically optimized metallic nanostructures specifically tailored for improved imaging performance.

## Nanophotonics in Biomedical Therapy and Imaging

### Nanophotonics in Therapy and Controlled Drug Delivery

**Fig. 3:**
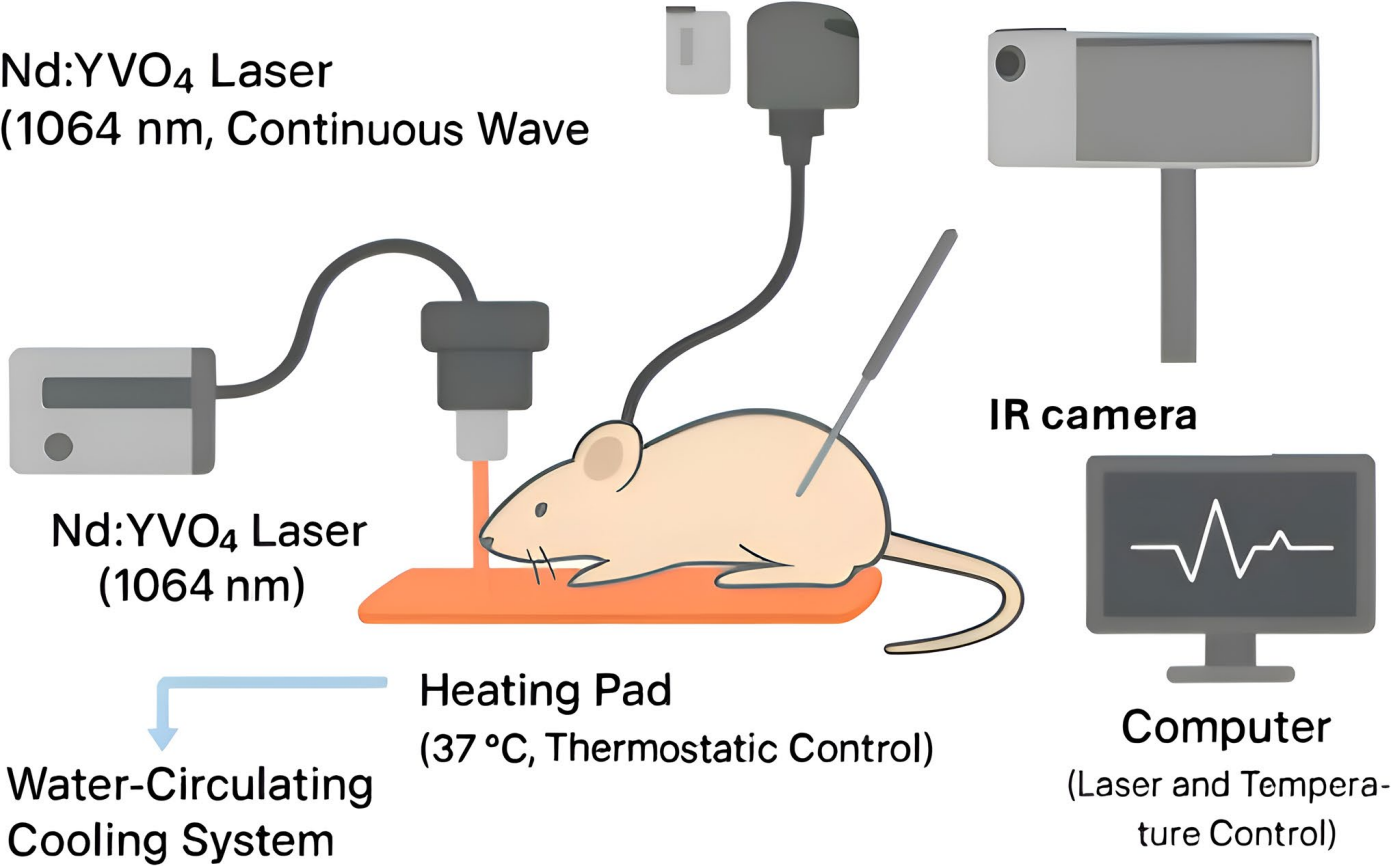
Schematic of the experimental setup for photothermal therapy, integrating controlled laser irradiation, animal anesthesia, and skin temperature regulation to ensure reproducible treatment conditions

Metallic nanostructures are central to PTT, where irradiation at plasmon resonance wavelengths converts light into localized heat, inducing hyperthermia to destroy cancer cells while sparing healthy tissue (Fig. 3). A major limitation is uneven heat distribution at tumor boundaries due to NIR attenuation, which can cause surface overheating. Strategies such as dual-laser irradiation, broadband sources like water-filtered infrared-A, and surface cooling methods (ice packs, gels, sprays, fluid-based cooling) have been developed to improve treatment safety [39, 44–46]. However, reproducibility of therapeutic outcomes remains a barrier, as nanoparticle distribution and heating efficiency vary considerably between studies and tumor models, complicating standardization and clinical translation.

Nanophotonics also enables optically controlled drug delivery, providing precise, on-demand therapy. Platforms such as MSNs, plasmonic liposomes, and gold nanocages employ light-responsive coatings or photodegradable triggers that enable controlled drug release with spatial and temporal precision. For example, gated MSNs incorporate photo-cleavable linkers or heat-sensitive polymers, while plasmonic liposomes undergo phase transitions upon NIR exposure, enabling site-specific chemotherapy with reduced systemic toxicity. Yet, inconsistent synthesis and functionalization protocols often result in variable release kinetics across laboratories, limiting reproducibility and comparability [45, 46].

Advanced carriers including exosomes, solid-lipid nanoparticles, nanospheres, and nanocapsules further enhance targeted delivery. Exosomes naturally deliver proteins and nucleic acids to specific cells, while nanocapsules with polymer shells provide stability and controlled release of lipophilic drugs. Polymersomes and nanofibers offer additional control over release kinetics, with electrospun nanofibers achieving tunable release profiles and polymersomes providing superior encapsulation [47]. Despite these advances, unresolved issues—such as variability in loading efficiency, incomplete understanding of long-term biodistribution, and limited scalability—restrict clinical adoption.

**Fig. 4:**
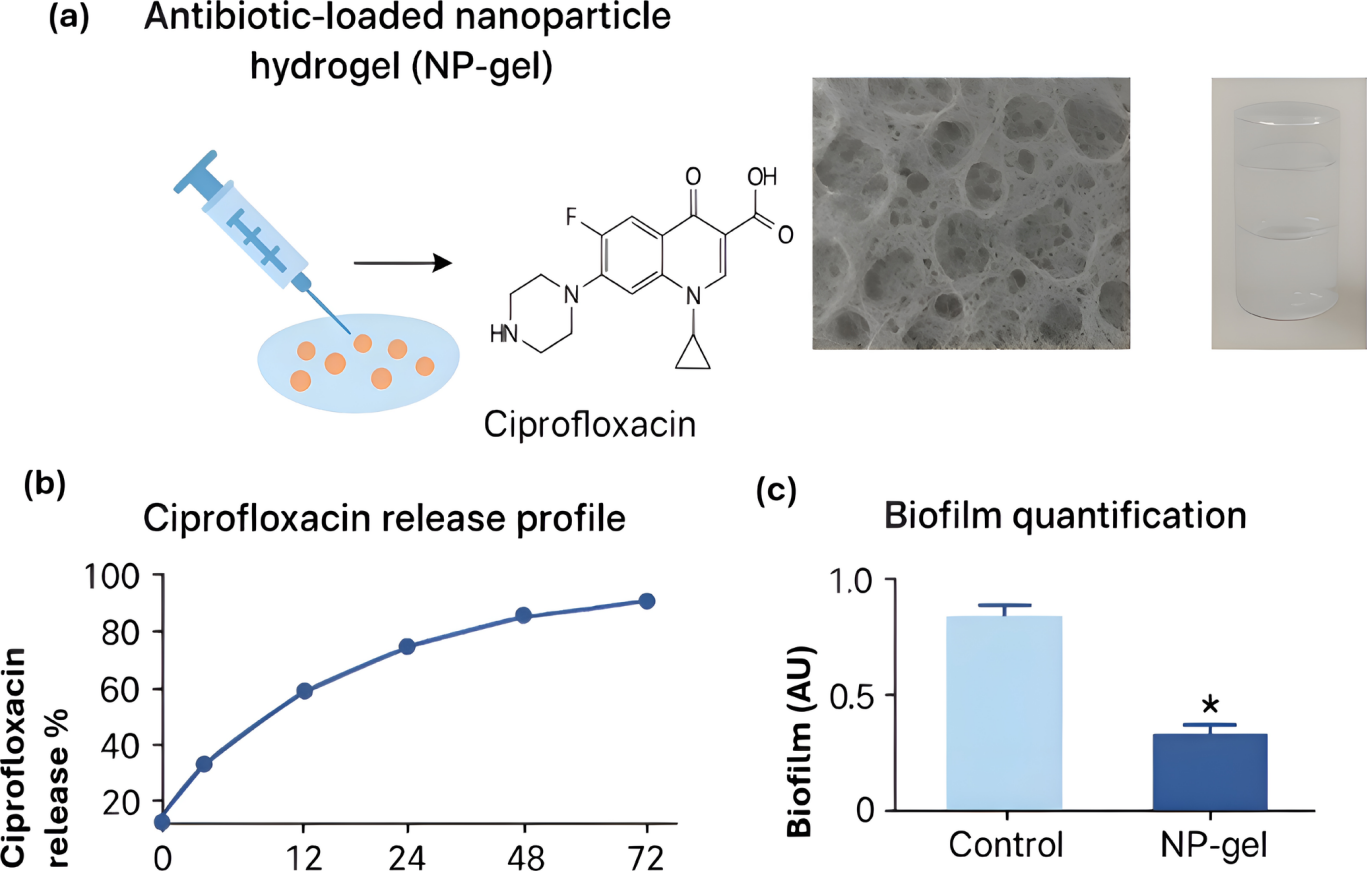
Antibiotic-loaded nanoparticle hydrogel (NP-gel) for localized, sustained antibiotic release. (**a**) Schematic representation, chemical structure, scanning electron microscopy (SEM) images, and photographs of NP-gel. (**b**) Ciprofloxacin release profile showing cumulative release kinetics. (**c**) Biofilm quantification following treatment, demonstrating significant inhibition of bacterial biofilm formation in preclinical models

In dental and orthopedic contexts, antimicrobial agents immobilized on micro- and nanomaterials enable localized, sustained release even under shear stress in the oral cavity. Nanoparticle–hydrogel systems (Fig. 4) have demonstrated shear-responsive antibiotic release. This has been shown to inhibit biofilm formation without tissue toxicity in preclinical models and shows promise for post-operative infection control [48]. Still, most studies remain preclinical, and variability in *in vivo* performance underscores the need for robust translational validation.

Beyond delivery, nanophotonics supports light-guided treatments and single-cell studies, enabling noninvasive diagnostics, selective drug targeting, and real-time drug tracking. Optical probes aid in mapping uptake, monitoring cytosolic interactions, and elucidating mechanisms underlying personalized therapies [49]. Nevertheless, widespread adoption is hindered by the complexity of device integration, risks of off-target effects, and regulatory uncertainty surrounding multifunctional nanoplatforms.

### Biomedical Imaging and Diagnostics

Nanophotonics has transformed biomedical imaging by enabling high-resolution, noninvasive visualization critical for early diagnosis. OCT, based on low-coherence interferometry, provides cross-sectional imaging with axial resolutions of 1–15 µm but often suffers from low contrast in homogeneous tissues. AuNPs enhance OCT as nanophotonic contrast agents via LSPR, generating strong optical scattering that amplifies signals in target areas and improves image reconstruction of tumor microstructures [50].

Plasmonic nanoprobes functionalized with antibodies or aptamers enable molecular-level diagnostics, positioning AuNP-based agents as promising tools for early detection and monitoring of malignancies, including hepatocellular carcinoma [51]. Super-resolution microscopy further overcomes the diffraction limit, achieving resolutions down to 10 nm using nonlinear methods such as Stimulated Emission Depletion and stochastic localization techniques (PALM, STORM, MINFLUX). Stranahan and Cang demonstrated near-nanometer localization of single molecules on SERS substrates, mapping plasmonic hotspots as small as 15 nm and correlating fluorescence maps with SEM images to inform nanostructure design [52].

**Fig. 5:**
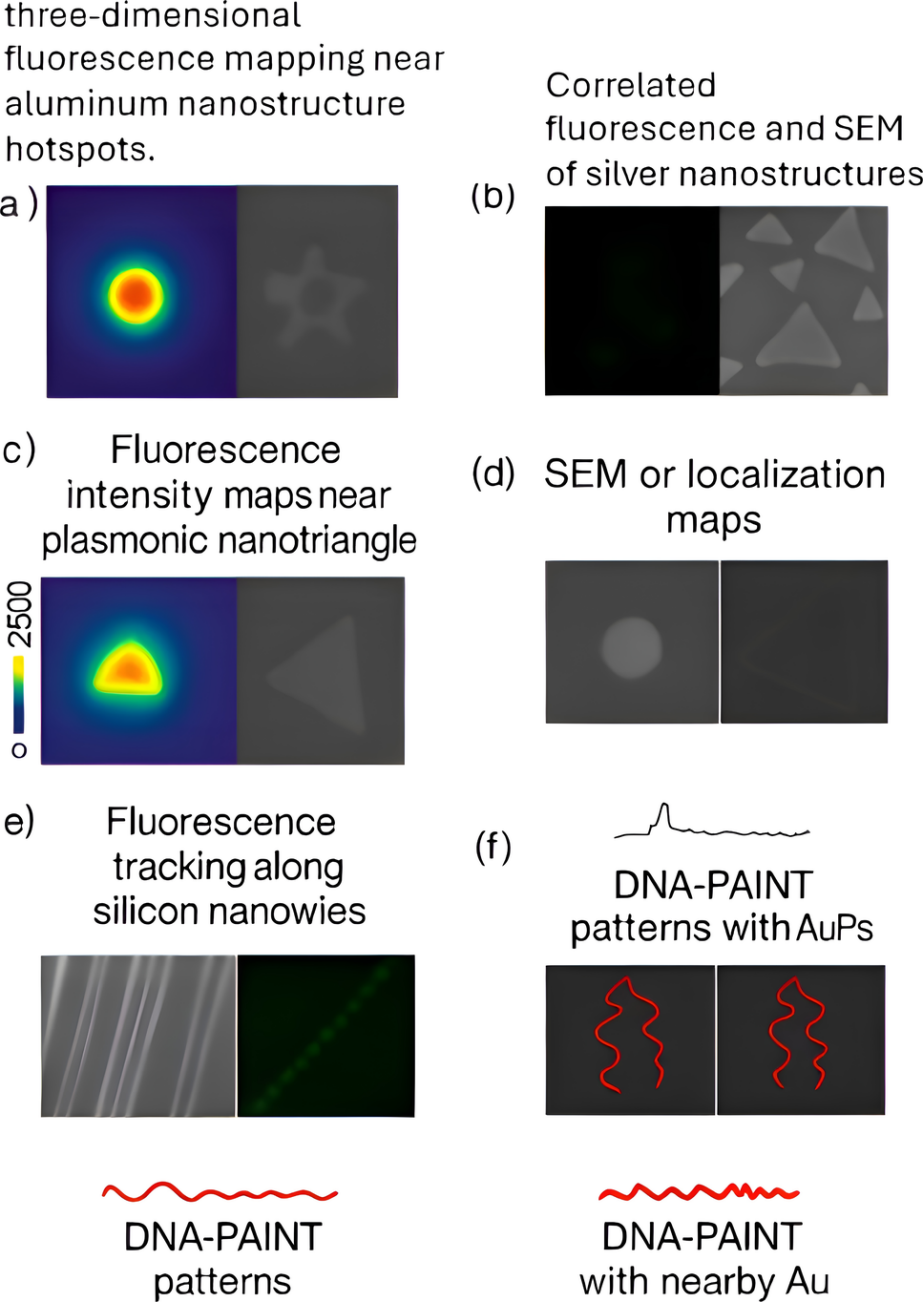
Single-molecule fluorescence probing of nanoscale light–matter interactions. (**a**) Super-resolved three-dimensional fluorescence mapping near aluminum nanostructure “hotspots.” (**b**) Correlated fluorescence and SEM of silver nanostructures. (**c**) Fluorescence intensity maps near plasmonic nanotriangles. (**d**) SEM and localization maps of aluminum nanodiscs. (**e**) Fluorescence tracking along silicon nanowires. (**f**) DNA points accumulation for imaging in nanoscale topography (DNA-PAINT) patterns with and without nearby gold nanoparticles (AuNPs), highlighting the effect of plasmonic enhancement on molecular localization precision

For practical diagnostics, portable SPR-based microfluidic platforms have been developed for rapid, multiplexed pathogen detection [53]. A compact device using a 705 nm LED with Kretschmann configuration and a CMOS sensor (Fig. 5) enabled real-time monitoring of *Staphylococcus aureus* and *Escherichia coli* with high specificity and sensitivity. This underscores the potential for POC applications [54].

Overall, nanophotonics continues to expand diagnostic capabilities, providing sensitive, scalable, and clinically adaptable tools. These advances lay the foundation for integration with biosensing and LOC platforms, explored in the following section.

## Biosensing and LOC Applications

Nanophotonic nanostructures are increasingly integrated into biosensing and lab-on-a-chip (LOC) systems, enabling ultrasensitive, portable diagnostic platforms for point-of-care (POC) testing. By exploiting strong light–matter interactions at the nanoscale, these materials enhance detection sensitivity, reduce reliance on labels or amplification, and allow real-time biomolecular analysis [51]. Plasmonic nanoparticles, photonic crystals, and nanostructured waveguides provide unique optical signatures, improving detection limits beyond conventional photonic devices.

Integrated photonic biosensors utilize micro- and nanoscale light-guiding elements—such as waveguides, resonators, and interferometers—where binding of target molecules alters refractive index, phase, or intensity signals. Nanophotonic enhancements, particularly through plasmonic field confinement and high-Q resonant cavities, significantly boost sensitivity by amplifying evanescent field interactions [52]. These advances directly complement microfluidic integration, which provides precise sample handling, reduced reagent consumption, and rapid analysis in compact device formats.

Recent innovations illustrate the synergistic role of nanophotonics and microfluidics in advancing LOC diagnostics. For example, CMOS-compatible silicon photonics and optofluidic photonic crystal platforms have been engineered for scalable, low-cost biosensing, while hybrid nanomaterials (e.g., silicon nitride combined with plasmonic coatings or polymers) improve both biocompatibility and sensitivity [55]. AI-driven signal processing further enhances data interpretation from complex optical responses, moving these systems closer to robust clinical deployment.

Representative examples highlight the diagnostic impact of these nanophotonic platforms. A lithographically fabricated nanophotonic biosensor integrated on glass demonstrated simultaneous light delivery, interaction, and detection within a compact chip. Using refractive index perturbations as the sensing mechanism, the device achieved detection limits as low as 14 ppm and a sensitivity of 139 fA/(g/dL) in liquid samples [56]. Similarly, portable SPR-based microfluidic systems leverage plasmonic nanostructures for multiplexed detection of bacterial infections. In one case, a miniaturized Kretschmann-configuration device employing a 705 nm LED and CMOS sensor enabled real-time monitoring of *Staphylococcus aureus* and *Escherichia coli* across clinically relevant ranges, demonstrating high specificity and applicability for decentralized infection management [53, 54].

Overall, nanophotonics plays a pivotal role in advancing biosensing and LOC technologies by enhancing optical sensitivity, enabling miniaturization, and providing material platforms compatible with scalable, point-of-care diagnostics. These innovations represent critical steps toward practical, decentralized healthcare and real-time environmental monitoring.

## Challenges and Limitations

Despite the immense promise of nanophotonics in biomedicine, several critical challenges must be addressed to enable safe and effective clinical translation. Importantly, many of these challenges are not only technical but also reproducibility- and adoption-related, highlighting the need for more rigorous and standardized evaluation across studies.

### Biocompatibility and Toxicity

The immune recognition, clearance, and long-term *in vivo* stability of nanophotonic materials remain incompletely understood. While gold nanoparticles (AuNPs) are often considered chemically inert, several studies have shown their preferential accumulation in reticuloendothelial organs such as the liver and spleen, leading to concerns about dose-dependent hepatotoxicity, oxidative stress, and chronic inflammation. For example, Cho et al. reported persistent AuNP retention in murine liver tissue for over 3 months, with signs of altered hepatic enzyme activity. Similarly, quantum dots have raised significant toxicity concerns due to the potential leakage of cadmium ions, which has been linked to renal dysfunction and cytotoxic effects in preclinical models [35, 36].

Surface modifications such as polyethylene glycol (PEG) or zwitterionic ligands can enhance circulation times and reduce protein corona formation. PEGylated AuNPs, for instance, demonstrated markedly reduced complement activation and extended blood half-life in rodent models. Zwitterionic coatings have also been shown to mitigate immune recognition *in vivo*, though comparative studies across different nanoparticle types remain limited. Importantly, while short-term *in vitro* assays often report minimal cytotoxicity, long-term *in vivo* studies reveal incomplete clearance of plasmonic nanostructures and potential for chronic tissue accumulation.

Overall, without large-scale, standardized toxicological studies that include chronic exposure models, establishing clinically safe thresholds for nanophotonic materials will remain difficult. These examples (Table 1) underscore the need for harmonized preclinical evaluation frameworks to support eventual regulatory approval [37, 38, 40].

**Table 1:** Biocompatibility and toxicity profiles of nanophotonic materials

Nanomaterial	Key Findings	Biocompatibility Strategies	Limitations	Ref
Gold nanoparticles (AuNPs)	Accumulate in liver and spleen via reticuloendothelial system (RES) clearance	PEGylation, zwitterionic surface coatings to reduce immune clearance	Long-term accumulation; chronic toxicity not fully understood	[36, 37]
Silver nanoparticles (AgNPs)	Antimicrobial but induce oxidative stress and cytotoxicity in hepatocytes	Surface capping with biopolymers (chitosan, PEG)	ROS generation, DNA damage, pro-inflammatory cytokine release	[38, 40]
Quantum dots (CdSe, CdTe)	High brightness for imaging but cadmium core leaches toxic ions	ZnS shell coating, polymer encapsulation	Long-term heavy metal release remains problematic	[41, 43]
Silica nanoparticles	Generally biocompatible at low doses; good drug-loading capacity	Surface silanization, PEGylation	High concentrations → lung inflammation, fibrosis risk	[42]
Carbon-based nanomaterials (CNTs, graphene)	Good photothermal properties; potential for neural interfacing	Oxidation, PEG functionalization to improve dispersibility	Pulmonary toxicity, granuloma formation after inhalation	[39, 44]
Upconversion nanoparticles (NaYF₄:Yb,Er)	Excellent deep-tissue imaging due to NIR excitation	Silica coating, phospholipid bilayer	Long-term renal clearance not well established	[45]
Polymeric nanoparticles (PLGA, PEG-PLA)	Biodegradable, FDA-approved carriers	Surface modification with targeting ligands	Burst release of payload; variable immune recognition	[46]

### Light Penetration in Biological Tissues

Limited light penetration due to scattering and absorption by tissue chromophores constrains clinical applications. Strategies to address this include using NIR-II/SWIR wavelengths (1000–1700 nm) for deeper tissue penetration and reduced scattering, intraluminal light delivery with optical fibers, and internally generated light from bioluminescent or chemiluminescent agents. While promising, most demonstrations remain at proof-of-concept stages, with limited evidence of reproducibility in larger animal models or patient cohorts. The translation of these methods will require not only technical optimization but also clinical-grade device integration and validation [28, 31].

### Stability and Reproducibility

Batch-to-batch variability in nanoparticle synthesis continues to impact optical performance and reliability. Even under similar laboratory conditions, small changes in reagent purity, reaction temperature, or storage stability can lead to marked differences in particle size, optical response, or biocompatibility. Environmental degradation, ligand desorption, and aggregation under physiological conditions further reduce effectiveness, raising concerns about reproducibility between studies. Currently, the absence of standardized synthesis protocols and quality benchmarks prevents meaningful comparison across laboratories, which undermines confidence in reported findings and slows regulatory acceptance [36, 47].

### Scale-up and Manufacturing

Scaling nanophotonic devices remains challenging due to complex fabrication, high production costs, and cleanroom requirements. While emerging roll-to-roll manufacturing, automated printing, and microfluidic synthesis approaches are being explored to reduce costs, most studies demonstrate feasibility at laboratory scale only. The lack of industrial-grade production pipelines limits reproducibility, cost-effectiveness, and availability for preclinical or clinical testing. Bridging this gap will require not only engineering innovations but also sustained industry partnerships to support scale-up [38, 39].

### Regulatory and Ethical Considerations

Bringing nanophotonics from the laboratory to the clinic presents challenges that go beyond the usual requirements for biomedical products. Many of these hurdles are tied to the unique nature of nanophotonic systems, which combine materials, light, and often digital components into a single platform. For example, a plasmonic nanoparticle designed for PTT cannot be evaluated in isolation—the associated laser hardware, beam geometry, and light–tissue dosimetry also need to be validated. This coupling makes it difficult to fit such systems neatly into existing regulatory categories of “drug” or “device,” often placing them in the more complex space of combination products [15, 43].

Another challenge is the lack of standardized optical performance benchmarks. While pharmacological agents are evaluated with established toxicity and efficacy assays, nanophotonics lacks common measures for parameters such as photothermal conversion efficiency, SERS enhancement factors, or quantum yield in biologically relevant media. As a result, studies often report impressive results that are difficult to reproduce or compare across laboratories. In parallel, biological interactions such as protein-corona formation and RES sequestration can alter biodistribution and clearance in unpredictable ways, raising questions about long-term retention and patient-to-patient variability [47, 48].

Ethical considerations are equally important. Platforms that integrate AI-enhanced nanosensors or cloud-based diagnostic tools open new opportunities for real-time health monitoring but also raise concerns about data privacy, algorithmic bias, and the security of patient information. These issues underscore the need for regulatory frameworks that are not only technically rigorous but also attentive to the societal implications of nanophotonics.

In short, nanophotonics faces a dual challenge: aligning with traditional biomedical safety requirements while also addressing its own modality-specific hurdles. Clearer regulatory pathways, shared optical benchmarks, and ethical safeguards will be essential if these technologies are to move beyond proof-of-concept demonstrations into reliable, clinically approved tools [28, 37].

## Future Perspectives and Emerging Trends

### Artificial Intelligence (AI) and Machine Learning (ML) Integration

AI and ML are reshaping nanophotonics by improving analytical, diagnostic, and predictive capabilities. In imaging, large datasets from OCT, fluorescence, and photoacoustic modalities train algorithms such as CNNs, SVMs, and generative models for automated tissue classification, with DL on reflectance confocal microscopy surpassing expert dermatologists in distinguishing skin lesions [36, 50, 54].

Beyond diagnostics, AI enables real-time adaptive feedback in theranostics, dynamically adjusting treatment parameters, while AI-enhanced nanosensors support personalized medicine through sensitive signal detection, continuous monitoring, and individualized treatment adjustments. ML further processes high-volume sensor data to improve accuracy, optimize designs, and, when integrated with IoT, enable remote health monitoring [15, 20].

Challenges include data scarcity, black-box models, system complexity, privacy concerns, adversarial risks, material variability, and regulatory barriers [50, 51]. Solutions will require explainable AI, robust data fusion, scalable computational frameworks, and stronger industry–academia collaboration. Demonstrations already show AI-enhanced nanotechnologies in imaging, tissue regeneration, and monitoring, with future directions emphasizing predictive modeling, data mining, and high-throughput testing to accelerate material discovery and drive personalized, adaptive nanophotonic healthcare [18, 23].

### Personalized Nanophotonics

The convergence of nanophotonics and personalized medicine offers a critical path for future impact. Patient-specific variations in pigmentation, density, vasculature, and chromophore levels influence light absorption, scattering, and fluorescence efficiency; pre-characterization with hyperspectral or photoacoustic imaging enables tailoring of diagnostic and therapeutic parameters [51]. In oncology, silica nanoparticles or gold nanorods tuned to the NIR window of a patient’s tumor can enhance penetration and efficacy while minimizing collateral damage [23, 31]. Integration of genomic and optical data with ML further optimizes nanoparticle size, shape, and surface chemistry for improved uptake and biodistribution, while personalized photothermal dosimetry and light-activated drug release support precise, individualized treatment plans. Collectively, personalized nanophotonics complements AI-driven systems to deliver diagnostics and therapies dynamically adapted to each patient’s biological and optical profile.

To provide a consolidated view of real-world biomedical implementations, Table 2 summarizes representative nanoplasmonic and related nanophotonic techniques, their detection mechanisms, and example applications with supporting references (Table 2).

**Table 2:** Biomedical applications and detection mechanisms of nanoplasmonic and related techniques

Technique	Biomedical Target	Detection Mechanism	Example application	Ref
Nanoplasmonic resonance (LSPR)	Cancer biomarker detection	Binding of biomolecules (HER2, PSA, etc.) to metallic nanostructures changes local refractive index, shifting plasmon resonance	HER2 detection in breast cancer serum	[1, 3, 20]
	Viral detection	Viral antigens interact with functionalized plasmonic chips → resonance peak shift	SARS-CoV-2 detection using plasmonic nanoparticle PCR platform	[22, 53]
	Bacterial pathogen detection	Antibody-coated plasmonic surfaces capture bacteria → measurable refractive index change	Multiplexed pathogen detection (*E. coli*) on portable SPR chips	[53, 54]
Lateral flow assays (LFAs)	Point-of-care diagnostics	Sample flows by capillary action, target captured by antibodies/aptamers on test lines; visible color or fluorescence readout	COVID-19 antigen test; hCG pregnancy test	[23, 25]
Fluorescence-based assays	Neurodegenerative biomarkers	Fluorescently labeled probes bind to amyloid-β/tau; signal intensity correlates with concentration	Alzheimer’s disease biomarker detection	[8, 9]
Electrochemical biosensors	Infectious disease diagnostics	Target binding alters current/impedance on electrode surface (EIS/voltammetry)	Mycobacterium tuberculosis DNA detection	[4, 21]
Colorimetric assays (AuNP-based)	Viral RNA detection	Target-induced aggregation of AuNPs changes solution color (red → blue)	Dengue virus RNA detection; DNA hybridization	[20, 21]
CRISPR-based diagnostics	Nucleic acid detection (viruses, cancers)	Cas enzyme activated by target → collateral cleavage of reporter probe → fluorescence or colorimetric readout	SHERLOCK/DETECTR for SARS-CoV-2	[22, 23]
Nanoparticle-enhanced assays	Cancer biomarker detection	Nanoparticles amplify optical/electrical signals by increasing surface area & local field	Glypican-3 (GPC3) detection in liver cancer	[7, 20]

Notably, several nanophotonic modalities have already entered clinical trials, underscoring their translational potential. Gold nanoshells have progressed into pilot clinical trials for photothermal therapy in prostate cancer [10], while SPR-based biosensors and LFAs are already cleared for routine diagnostics, including pregnancy and COVID-19 antigen testing. By contrast, most nanoprobe-based *in vivo* imaging techniques remain preclinical, and quantum dots in particular face persistent regulatory concerns regarding toxicity and long-term clearance. These distinctions highlight the importance of coupling performance advances with rigorous clinical evaluation and regulatory alignment.

Beyond these specific examples, it is also important to consider the comparative clinical status of different nanophotonic modalities. As summarized in Table 3, while plasmonic AuNPs have advanced into early clinical trials with promising photothermal and imaging results, issues of long-term accumulation highlight the gap between experimental efficacy and regulatory acceptance. In contrast, quantum dots remain largely preclinical due to toxicity concerns despite their strong optical stability [57–61]. This comparative perspective underscores the uneven progress across modalities and highlights both opportunities and translational barriers that must be addressed for clinical adoption (Table 3).

**Table 3:** Comparative clinical status, advantages, and limitations of representative nanophotonic modalities

Modality	Clinical status	Advantages	Limitations	Ref
Metamaterials/Nanoantennas	Research stage	Tunable optical properties, potential for miniaturization	No clinical translation yet	[57, 58]
Optical (NIR-I, II)	Mostly preclinical	High sensitivity, non-ionizing radiation	Limited penetration depth (< 2 cm), scattering	[57, 59]
Plasmonics (Au NPs)	Early clinical trials	Strong photothermal conversion, tunable optical response	Long-term accumulation, clearance issues	[58, 60]
Quantum dots	Preclinical	Bright, stable fluorescence	Biocompatibility and potential toxicity	[59, 61]

### Outlook

Looking ahead, the translation of nanophotonics into clinical practice will depend on coordinated progress across several fronts. Table S1 provides a comparative overview of major nanophotonic platforms, contrasting their performance, maturity, and translational status [62–67]. This highlights the uneven progress across modalities—for example, plasmonic nanostructures advancing into pilot clinical trials, while quantum dots and metamaterials remain largely preclinical. On the research side, there is a clear need to design biocompatible and, where appropriate, transient or stimuli-responsive nanophotonic platforms that reduce long-term toxicity risks. Expanding work into the NIR-II/SWIR window will also be important, as this can help overcome tissue penetration barriers and unlock deeper imaging capabilities. At the same time, the integration of AI and ML should move beyond proof-of-concept demonstrations, focusing on explainable, data-efficient models that can be trusted in real-world biomedical settings [14].

Regulatory and translational pathways will also play a defining role. Standardized synthesis and characterization protocols are essential to ensure reproducibility, while systematic studies of toxicology, pharmacokinetics, and biodistribution under physiologically relevant conditions will build confidence in clinical safety. Early and ongoing dialogue with regulatory agencies can help align device development with approval requirements and shorten the path from laboratory to bedside [44, 50].

Finally, success in this field will depend on collaboration that extends beyond traditional disciplinary boundaries. Materials scientists, engineers, clinicians, and computational experts must work together to accelerate innovation, while academia–industry partnerships can provide the scale and infrastructure needed for manufacturing and clinical trials. On a global level, stronger international collaboration will be important to harmonize regulatory standards and facilitate data-sharing, ensuring that advances in nanophotonics can benefit patients worldwide [35].

## Summary

This review outlined how nanophotonics is advancing molecular imaging and photobiology through nanoscale light–matter interactions, fabrication strategies, and applications in super-resolution imaging, targeted phototherapies, and LOC biosensing. Integration with AI and ML enables real-time image analysis and adaptive feedback, enhancing diagnostic precision and therapeutic monitoring. Challenges such as biocompatibility, limited light penetration, stability, and scalability persist, but emerging trends in personalized nanophotonics promise patient-specific imaging and therapy. Overall, nanophotonics is poised to drive precision diagnostics and minimally invasive, image-guided treatments in molecular medicine.

## Conclusion

Nanophotonics is transforming molecular imaging and photomedicine by enabling high-resolution imaging, targeted phototherapies, and ultrasensitive biosensors that interrogate biological processes at cellular and molecular scales. These advances are beginning to influence preclinical and translational research with strong clinical potential. Integration with AI and ML further extends these capabilities, allowing real-time image analysis, predictive modeling, and adaptive, patient-specific treatment strategies that strengthen the precision and impact of nanophotonic systems.

Despite this progress, key hurdles remain, including biocompatibility, limited light penetration, reproducibility in nanostructure synthesis, and regulatory complexities. Addressing them will require sustained interdisciplinary collaboration across materials science, photonics, imaging, engineering, medicine, and data science. Looking ahead, personalized nanophotonics promises diagnostics and therapies tailored to individual molecular profiles, aligning with the goals of precision medicine. With expanding research and clinical partnerships, nanophotonics is poised to move from laboratory innovation to clinical reality, advancing precision, effectiveness, and accessibility in molecular diagnostics and therapies worldwide.

## Supplementary Information

Below is the link to the electronic supplementary material.

ESM 1 (DOCX 15.6 KB)

## Data Availability

Not applicable.
