# Supplementary material for: Nanophotonics in Molecular Imaging and Biomedicine: Diagnostics, Therapies, and Translational Challenges
Source: Mol Imaging Biol. 2025 Nov 17;27(6):914–29. doi: 10.1007/s11307-025-02061-w (PMC12804207; doi:10.1007/s11307-025-02061-w)
Supplement: Supplementary file 1 — (DOCX 15.6 KB) [file 11307_2025_2061_MOESM1_ESM.docx]

**Table S1. Comparative performance, maturity, and translational status of nanophotonic platforms**

| Platform | Performance( Strength/ constraints ) | Maturity | Translational status | References |
| --- | --- | --- | --- | --- |
| Plasmonics (Au nanoshells/rods; SERS tags) | High optical cross-sections; strong photothermal effect; molecular specificity. Limitation: shallow penetration, RES accumulation. | Late preclinical to early clinical | Pilot clinical trial with gold nanoshell photothermal therapy; SERS explored for cancer diagnostics. | [62,63] |
| Quantum dots (QDs) | Bright, narrow emission; multiplexing; NIR-II improves depth. Limitation: toxicity, long-term clearance. | Mostly preclinical. | Preclinical imaging and tracking; carbon/silicon QDs under investigation for reduced toxicity. | [64] |
| Metamaterials | Tailored EM response; boost MRI SNR; tunable optical/magnetic behavior. Limitation: integration, reproducibility. | Prototype stage | Demonstrated metamaterial-enhanced MRI coils; not in routine clinical use. | [65] |
| Photonic crystals (PhC) biosensors | High-Q resonances sensitive label-free detection; integrable. Limitation: thermal drift, optical readout | Extensive lab/POC demos | Biosensors for proteins, metabolites, cancer biomarkers. | [66] |
| Nanoantennas | extreme near-field enhancement; potential single-molecule detection. Limitation: fabrication reproducibility, stability | Early research | Concept studies for integrated sensing; no clinical translation yet. | [67] |
